# *Helicobacter pylori* and metabolic-associated fatty liver disease severity: a meta-analysis

**DOI:** 10.3389/fmed.2026.1821076

**Published:** 2026-06-17

**Authors:** Lingyun Zhang, Yuexia Zhang, Lei Qiao, Ruihan Wu, Feng He, Yuxin Guan, Lei Li

**Affiliations:** 1 Department of Gastroenterology, Beijing Jishuitan Hospital, Capital Medical University, Beijing, China; 2 Health Management Center, Beijing Jishuitan Hospital, Capital Medical University, Beijing, China

**Keywords:** *Helicobacter pylori*, meta-analysis, metabolic-associated fatty liver disease, severity of illness index, study

## Abstract

**Background and aims:**

Metabolic-associated fatty liver disease (MAFLD) is a global health concern. Observational evidence has suggested a possible relationship between *Helicobacter pylori* infection and MAFLD severity; however, severity-focused data remain inconsistent and methodologically heterogeneous.

**Methods:**

We searched the international databases PubMed, Embase and Web of Science and the Chinese databases China National Knowledge Infrastructure, Chinese Scientific Journal Database (VIP) and Wanfang between January 2015 and December 2024. Observational studies reporting *H. pylori* status and MAFLD/non-alcoholic fatty liver disease (NAFLD) severity were included. Studies using NAFLD criteria were considered clinically comparable with MAFLD because they were conducted before the broad adoption of the MAFLD terminology and enrolled fatty liver disease populations defined by hepatic steatosis with metabolic features. Pooled odds ratios (ORs) were calculated using random-effects models with restricted maximum likelihood estimation and Hartung–Knapp adjustment.

**Results:**

Seven studies, comprising 11,168 participants (4,980 *H. pylori*-positive; 6,188 *H. pylori*-negative), were included. The overall random-effects analysis did not demonstrate a statistically significant association between *H. pylori* infection and MAFLD severity (pooled OR 1.47, 95% confidence interval [CI]: 0.83–2.59, *p* = 0.148*; τ*^2^ = 0.1325), with substantial heterogeneity (*I*^2^ = 79.0%). In the non-invasive assessment subgroup, the pooled estimate was OR = 1.22 (95% CI: 0.95–1.57). The two biopsy-based studies showed high but highly imprecise individual estimates and were not interpreted as evidence of a stronger association. Geographic analysis showed an association only in Chinese populations (OR = 1.31, 95% CI: 1.15–1.49, *I*^2^ = 0%).

**Conclusion:**

Current evidence does not demonstrate a statistically significant overall association between *H. pylori* infection and MAFLD severity. A modest association was observed in Chinese studies, although biopsy-based evidence remains too imprecise for inference. Large-scale prospective studies with standardized assessment of active *H. pylori* infection and MAFLD severity are needed before causal or therapeutic conclusions can be drawn.

## 1 Introduction

Metabolic-associated fatty liver disease (MAFLD), previously known as non-alcoholic fatty liver disease (NAFLD), represents a spectrum of liver conditions characterized by hepatic steatosis in the presence of metabolic dysfunction (1). The nomenclature change from NAFLD to MAFLD in 2020 reflected a paradigm shift toward positive diagnostic criteria based on metabolic risk factors rather than the exclusion of alcohol consumption (2). Metabolic-associated fatty liver disease is diagnosed by the presence of hepatic steatosis plus one of the following: overweight/obesity, type 2 diabetes mellitus or evidence of metabolic dysregulation (3). This condition has become the most common chronic liver disease worldwide, affecting approximately one-quarter of the global population, with prevalence rates ranging from 20 to 40% depending on geographic region and population characteristics (4).

The clinical spectrum of MAFLD ranges from simple steatosis to metabolic-associated steatohepatitis (MASH, formerly NASH), which is characterized by hepatocellular injury, inflammation and varying degrees of fibrosis (5). Among patients with MAFLD, approximately 20–30% develop MASH, and a subset progress to cirrhosis over time (6). Fibrosis stage is one of the strongest predictors of liver-related morbidity and mortality in fatty liver disease, making the identification of factors associated with disease severity clinically important (7).

*Helicobacter pylori* is a gram-negative bacterium that chronically infects the gastric mucosa of more than half of the world’s population (8). Although *H. pylori* is primarily recognized for its role in peptic ulcer disease and gastric cancer, accumulating evidence suggests that this infection may be associated with extragastric manifestations, including metabolic disorders (9). Several mechanisms have been proposed to link *H. pylori* infection with metabolic dysfunction, including chronic low-grade systemic inflammation, insulin resistance, altered secretion of gastric hormones and dysregulation of adipokines (10, 11). These pathophysiological pathways overlap with mechanisms implicated in MAFLD development and progression, but they do not establish causality.

Recent observational studies and meta-analyses have investigated the relationship between *H. pylori* infection and the prevalence of NAFLD/MAFLD, with many reporting positive associations (12, 13). A 2024 meta-analysis by Zhang et al., which included 34 studies with 175,575 individuals, reported that *H. pylori* infection was associated with a higher incidence of NAFLD (14). However, prevalence-focused analyses do not directly address disease severity, which is a more clinically relevant outcome because fibrosis and steatohepatitis drive progression to cirrhosis, liver failure and hepatocellular carcinoma (15).

Despite growing interest in the *H. pylori*–MAFLD relationship, uncertainty remains regarding whether *H. pylori* infection is associated with more severe forms of MAFLD. Previous reviews have noted that only a minority of studies evaluated *H. pylori* prevalence according to NAFLD severity, and severity definitions varied substantially across histological, elastographic, biochemical and imaging-based approaches. This variability may affect both the magnitude and interpretability of pooled estimates.

Therefore, we conducted this systematic review and meta-analysis to evaluate the association between *H. pylori* infection and MAFLD severity, as assessed by liver biopsy, non-invasive fibrosis scores, transient elastography or imaging-based grading systems. The analysis was designed to quantify the overall association while explicitly assessing whether heterogeneity in severity assessment method, infection ascertainment and geographic region limited the interpretation of pooled results.

## 2 Methods

### 2.1 Search strategy and data sources

A comprehensive systematic literature search of multiple electronic databases, including the international databases PubMed, Embase and Web of Science and the Chinese databases China National Knowledge Infrastructure (CNKI), Chinese Scientific Journal Database (VIP) and Wanfang Data, was conducted using data from January 2015 to December 2024. The date restriction was selected because contemporary MAFLD/NAFLD severity studies using biopsy, transient elastography, controlled attenuation parameter (CAP) and standardized non-invasive scoring became more consistently available during this period, and the earliest eligible severity-focused study identified in the final dataset was published in 2015. The search strategy combined Medical Subject Headings (MeSH) terms and free-text words related to (1) *H. pylori* infection: ‘*Helicobacter pylori’*, ‘*H. pylori*’, ‘HP’, ‘*Helicobacter*’ and ‘*Helicobacter pylori* infection’; (2) fatty liver disease: ‘nonalcoholic fatty liver disease’, ‘NAFLD’, ‘metabolic associated fatty liver disease’, ‘MAFLD’, ‘metabolic dysfunction-associated fatty liver disease’, ‘nonalcoholic steatohepatitis’, ‘NASH’, ‘MASH’, ‘hepatic steatosis’ and ‘fatty liver’; and (3) disease severity: ‘severity’, ‘fibrosis’, ‘cirrhosis’, ‘steatohepatitis’, ‘liver stiffness’, ‘CAP’, ‘controlled attenuation parameter’ and ‘NAS score’. The search was limited to studies published in English or Chinese. Japanese and Korean databases were not searched directly; however, English-language reports from Japan and Korea were captured through the international databases. Records retrieved from CNKI, VIP and Wanfang were de-duplicated by title, author, year, journal, digital object identifier and study population before screening. For example, the search strategy in PubMed was: (((*Helicobacter pylori*[MeSH Terms]) OR (‘*H. pylori*’ [Title/Abstract])) AND ((Metabolic associated fatty liver disease[Title/Abstract]) OR (‘NAFLD’[Title/Abstract])) AND ((Severity of Illness Index[MeSH Terms]) OR (‘fibrosis’[Title/Abstract]))). Reference lists of relevant articles and previous systematic reviews were manually screened to identify additional eligible studies. The review has been registered in INPLASY (registration number: INPLASY202650080); the search strategy, eligibility criteria and statistical plan were defined before full-text data extraction. Reporting followed the PRISMA 2020 statement (16).

### 2.2 Inclusion and exclusion criteria

Studies were eligible for inclusion if they met the following criteria: (1) the study population included adults (aged ≥18 years) with MAFLD/NAFLD diagnosed by liver biopsy, imaging (ultrasound, computed tomography, magnetic resonance imaging) or transient elastography; (2) *H. pylori* infection status was determined using validated methods, including 13C or 14C urea breath test (UBT), gastric mucosal biopsy histology, stool antigen test or serum *H. pylori* immunoglobulin G antibodies; (3) studies reported at least one outcome measure related to MAFLD severity, including histological severity (NASH vs. NAFLD, fibrosis stage, steatosis grade, inflammation grade), non-invasive fibrosis scores (NAFLD fibrosis score, fibrosis-4 score, AST to platelet ratio index, BARD score) or imaging-based severity assessments (ultrasound grading of mild/moderate/severe steatosis, CAP values or liver stiffness measurements); (4) studies provided sufficient data to calculate odds ratios (ORs), risk ratios or hazard ratios with 95% confidence intervals (CIs) or raw data to construct 2 × 2 contingency tables; (5) study designs included cross-sectional studies, cohort studies or case–control studies; and (6) articles were published as full-text original research papers in peer-reviewed journals between 2015 and 2024. Studies using NAFLD criteria were included and considered clinically comparable with MAFLD because most eligible studies predated the MAFLD nomenclature and because their participants had hepatic steatosis assessed in populations enriched for metabolic risk. For harmonization, severe MAFLD/NAFLD was operationally defined as NASH or advanced histological features in biopsy studies, moderate-to-severe steatosis on ultrasound, elevated CAP or elevated liver stiffness/fibrosis threshold in non-invasive studies. The exclusion criteria were as follows: (1) review articles, meta-analyses, editorials, commentaries, case reports or conference abstracts without full-text availability; (2) studies that only reported MAFLD/NAFLD prevalence without information on disease severity; (3) animal studies or *in vitro* experiments; (4) studies with overlapping or duplicate populations, for which the study with the largest sample size or most complete severity data was retained; and (5) studies lacking essential data for meta-analysis after data extraction.

### 2.3 Study selection and data extraction

Two independent reviewers screened titles and abstracts of all retrieved articles based on the predefined inclusion and exclusion criteria. Full-text articles of potentially eligible studies were then obtained and independently assessed for final inclusion. Disagreements were resolved through discussion with a third reviewer until a consensus was reached. Data extraction was performed independently by two reviewers using a standardized data extraction form. The following information was collected from each included study: (1) study characteristics – first author, publication year, country of origin, study design, sample size and setting; (2) participant characteristics – age, sex distribution, body mass index and prevalence of metabolic comorbidities when available; (3) *H. pylori* detection method and positivity rate, including whether the method primarily reflected active infection or serological evidence of exposure; (4) MAFLD/NAFLD diagnosis and severity assessment methods; (5) outcomes – number of participants with severe vs. mild disease in *H. pylori*-positive and *H. pylori*-negative groups or mean values with standard deviations for continuous severity measures; (6) adjusted or unadjusted effect estimates when available; and (7) covariates adjusted for in multivariable analyses. Information on proton pump inhibitor use, prior eradication therapy and the time interval between *H. pylori* testing and liver severity assessment was extracted when reported.

### 2.4 Quality assessment

The methodological quality of included observational studies was assessed using the Newcastle–Ottawa Scale (NOS), which evaluates three domains: selection of study groups (4 points), comparability of groups (2 points) and ascertainment of exposure or outcome (3 points), with a maximum score of 9 points (17). Studies scoring 7–9 points were considered high quality, 4–6 points as moderate quality and 0–3 points as low quality. Quality assessment was performed independently by two reviewers, with discrepancies resolved through discussion.

### 2.5 Statistical analysis

Statistical analyses were performed using R statistical software (version 4.0 or higher) with the ‘meta’ and ‘metafor’ packages. For binary outcomes (severe vs. nonsevere MAFLD/NAFLD), pooled ORs with 95% CIs were calculated. Because a small number of clinically heterogeneous observational studies was expected, the primary random-effects model used restricted maximum likelihood estimation for *τ*^2^ with Hartung–Knapp adjustment for CIs. Statistical heterogeneity was quantified using Cochran’s Q test, *τ*^2^ and the *I*^2^ statistic, and *I*^2^ values of 25, 50 and 75% were interpreted as representing low, moderate and high heterogeneity, respectively. The overall pooled estimate was treated as descriptive because biopsy-based and noninvasive studies assessed related but not identical constructs of severity. Subgroup analyses were performed by method of severity assessment and geographic region. Biopsy-based studies were presented narratively rather than interpreted as a stable subgroup pooled estimate because only two small studies were available and the CI was extremely wide. Subgroup tests were interpreted cautiously and considered hypothesis-generating. Sensitivity analysis used the leave-one-out method, whereby the meta-analysis was repeated after sequentially excluding each study to determine whether the direction of effect materially changed. Meta-regression was not performed because <10 studies were available, and formal tests for funnel-plot asymmetry, including Egger’s test and trim-and-fill, were not performed because such methods have low power and can be misleading with <10 studies (18).

All statistical tests were two-sided, and a *p*-value of <0.05 was considered statistically significant for pooled effect estimates. As this meta-analysis included only observational studies, randomized trials of *H. pylori* eradication were not eligible for the association meta-analysis; available trials were considered separately in the Discussion section as intervention evidence.

## 3 Results

### 3.1 Literature search and study selection

**Figure 1:**
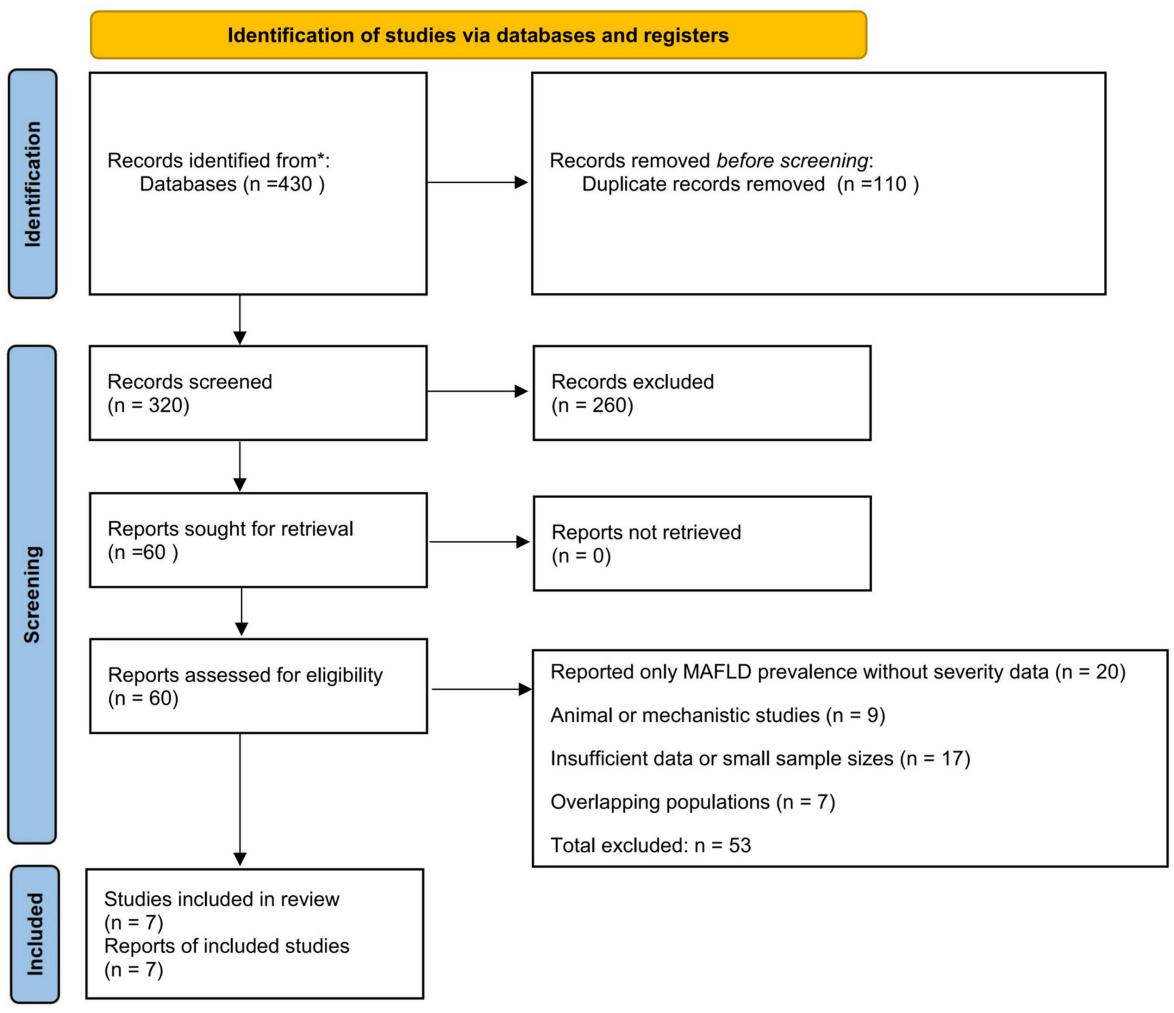
PRISMA flow diagram.

The initial systematic literature search identified approximately 430 potentially relevant articles from all databases. After removing 110 duplicate records, including duplicate records across CNKI, VIP and Wanfang Data, 320 unique citations remained for title and abstract screening. Of these, 260 articles were excluded based on a title and abstract review because they were unrelated to MAFLD/NAFLD, were review articles or case reports, or clearly lacked information on disease severity. The remaining 60 articles underwent full-text evaluation. Following a detailed assessment, 53 articles were excluded for the following reasons: 20 studies reported only MAFLD/NAFLD prevalence without severity data, 9 were animal or mechanistic studies, 17 had insufficient data or small sample sizes and 7 had overlapping populations with other included studies. Ultimately, 7 studies met all inclusion criteria and were included in the quantitative meta-analysis (Figure 1).

### 3.2 Characteristics of included studies

The seven included studies, published between 2015 and 2021, comprised a total of 11,168 participants, including 4,980 *H. pylori*-positive individuals and 6,188 *H. pylori*-negative individuals (Table 1) (19–25).

**Table 1:** Characteristics of included studies.

Study	Country	Study design	Population	Hp detection	MAFLD severity assessment	Hp + (n)	Hp-(n)	References
Sumida, 2015	Japan	Case–control Study	Biopsy-proven NAFLD patients from gastroenterology clinic	Serum anti-*H. pylori* IgG (serology)	Liver biopsy; NASH vs. non-NASH (Matteoni type 3–4 vs. 1–2)	52	78	(19)
Doulberis, 2020	Switzerland / Greece	Cross-sectional Study	Morbidly obese patients undergoing bariatric surgery	Gastric biopsy RUT + histology (active infection)	Liver biopsy; NASH vs. NAFL (FLIP algorithm)	15	49	(20)
Chen, 2020	China	Cross-sectional Study	NAFLD patients diagnosed by ultrasound	UBT and/or *H. pylori* antibody (mixed)	Ultrasound grading; moderate+severe vs. mild NAFLD	47	44	(21)
Han, 2021	Korea	Cross-sectional Study	Health check-up cohort assessed by FibroScan	Serum anti-*H. pylori* IgG (serology)	Transient elastography CAP; CAP ≥268 vs. < 268 dB/m	708	1,076	(22)
Yan, 2021	China	Cross-sectional Study	Health examinees, NAFLD diagnosed by ultrasound	13C-UBT (active infection)	Ultrasound-defined NAFLD severity grading	464	721	(23)
Xu, 2020	China	Cross-sectional Study	Health check-up cohort, NAFLD diagnosed by ultrasound	Serum anti-*H. pylori* IgG/IgM (serology)	Ultrasound grading; moderate+severe vs. mild NAFLD	2,516	2,309	(24)
Liu, 2021	China	Cross-sectional Study	Health check-up men, NAFLD by ultrasound and TE	13C-UBT (active infection)	Transient elastography; LSM ≥ 7.4 vs. < 7.4 kPa	1,178	1911	(25)

Hp, *Helicobacter pylori*; NAFLD, non-alcoholic fatty liver disease; MAFLD, metabolic-associated fatty liver disease; NASH, non-alcoholic steatohepatitis; NAFL, non-alcoholic fatty liver; UBT, urea breath test; RUT, rapid urease test; CAP, controlled attenuation parameter; TE, transient elastography; LSM, liver stiffness measurement; FLIP, fatty liver inhibition of progression. Serology may reflect prior exposure rather than active infection.

**Figure 2:**
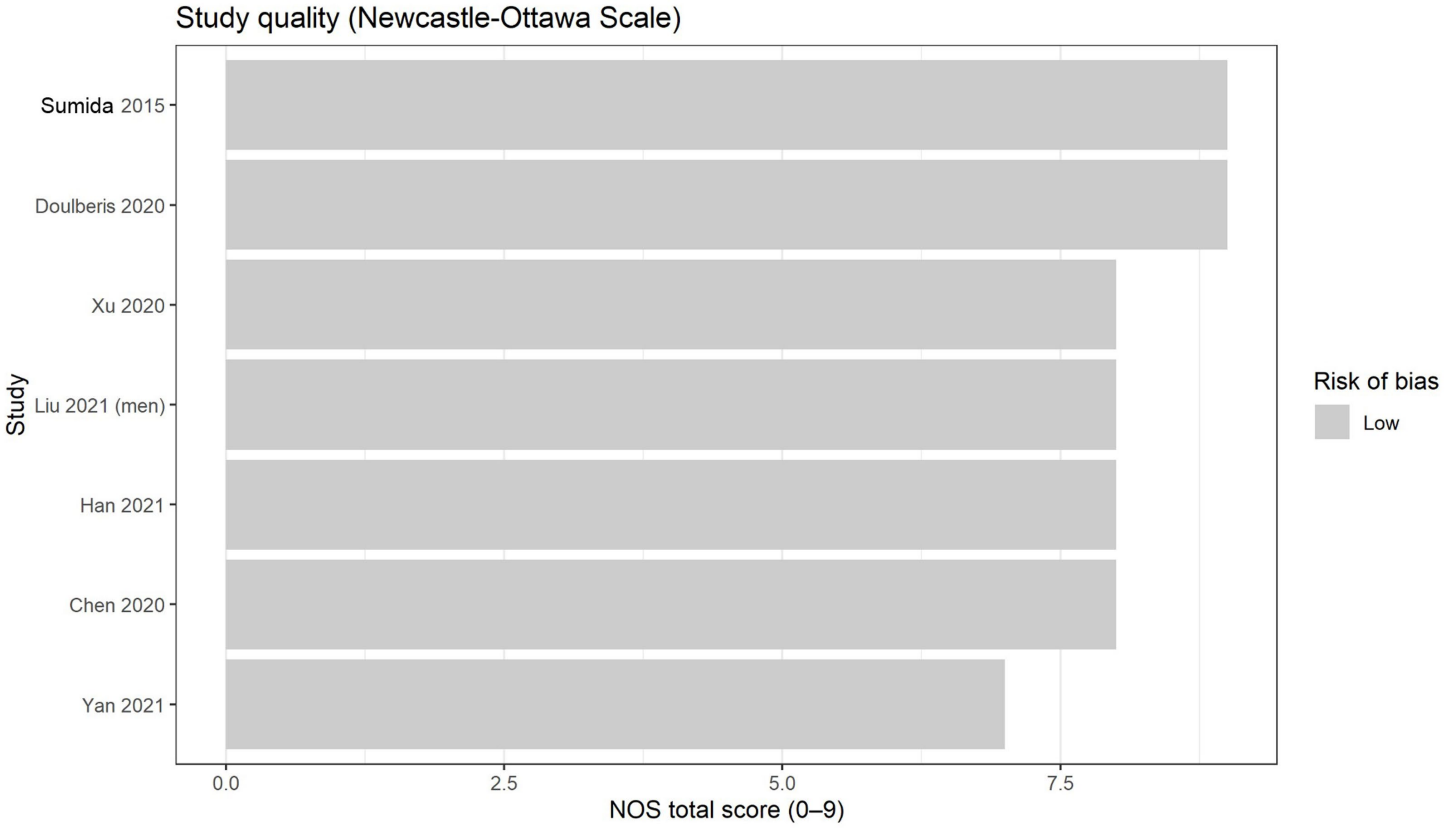
Quality assessment of included studies using the Newcastle-Ottawa scale. All seven studies demonstrated moderate to good methodological quality, with NOS scores ranging from 6 to 9 points. The horizontal bars represent the total NOS score for each study.

Four studies were conducted in China (21, 23–25), one in Japan (19), one in Korea (22) and one in Switzerland/Greece (20). Regarding study design, six were cross-sectional or observational studies (20–25), and one was a case–control study involving patients with biopsy-proven NAFLD (19). The *H. pylori* detection methods varied: three studies used 13C or 14C UBTs as active infection tests (21, 23, 25), one used gastric biopsy with rapid urease testing and histology (20) and three used serological anti-*H. pylori* antibodies or mixed serological/UBT methods (19, 22, 24). For MAFLD/NAFLD severity assessment, two studies utilized liver biopsy (19, 20), and five employed non-invasive methods: ultrasound grading (21, 23, 24), transient elastography with CAP (22) or liver stiffness measurement (25). The quality assessment using the NOS revealed that all seven studies were of moderate to good quality (Figure 2), with NOS scores ranging from 6 to 9 points.

### 3.3 Association between *Helicobacter pylori* infection and MAFLD severity

**Figure 3:**
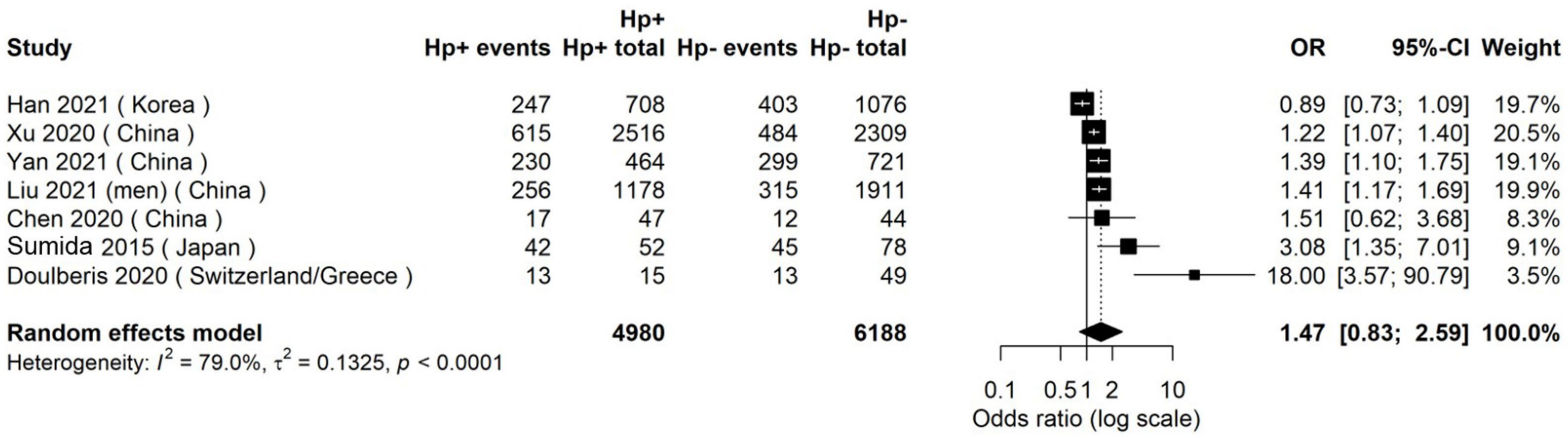
Overall forest plot showing the association between *H. pylori* infection and MAFLD/NAFLD severity. The pooled analysis of 7 studies yielded an odds ratio of 1.47 (95% CI 0.83–2.59, *p* = 0.148) with substantial heterogeneity (*I*^2^ = 79.0%, τ^2^ = 0.1325). The overall pooled estimate should be interpreted cautiously because biopsy-based and non-invasive studies assessed related but not identical severity constructs.

The overall meta-analysis of seven studies did not demonstrate a statistically significant association between *H. pylori* infection and MAFLD/NAFLD severity, with a pooled OR of 1.47 (95% CI: 0.83–2.59, *p* = 0.148) (Figure 3). Substantial heterogeneity was observed across studies (*I*^2^ = 79.0%, *τ*^2^ = 0.1325, *p* < 0.0001), indicating considerable variation in effect sizes. Visual inspection of the forest plot revealed that effect estimates ranged from OR = 0.89 (95% CI: 0.73–1.09) in the study by Han et al. (22) to OR = 18.00 (95% CI: 3.57–90.79) in the study by Doulberis et al. (20), reflecting major differences in study populations, geographical regions, *H. pylori* detection methods and MAFLD/NAFLD severity assessment methods.

### 3.4 Subgroup analyses

#### 3.4.1 Subgroup analysis by assessment method

**Figure 4:**
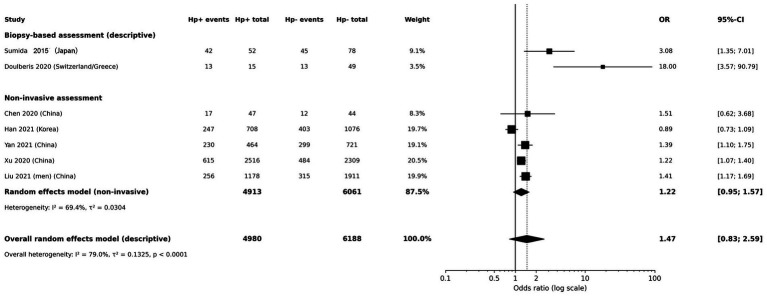
Forest plot of the association between *H. pylori* infection and MAFLD/NAFLD severity stratified by assessment method. Biopsy-based studies are displayed individually because the very small number of studies and the extremely wide confidence interval make a biopsy subgroup pooled estimate unstable. The non-invasive subgroup pooled estimate was OR 1.22 (95% CI 0.95–1.57).

To explore sources of heterogeneity, subgroup analysis was performed based on the method used to assess MAFLD/NAFLD severity (Figure 4). The two biopsy-based studies were not interpreted as providing a stable pooled biopsy estimate because they included only 194 participants, had markedly different effect sizes and produced an extremely wide CI. The individual biopsy study estimates were OR = 3.08 (95% CI: 1.35–7.01) for the study by Sumida (19) and OR = 18.00 (95% CI: 3.57–90.79) for the study by Doulberis et al. (20). The extremely wide CI generated when these two studies were pooled indicates limited precision and instability of the estimate; therefore, these data do not support the conclusion that the association is stronger when assessed by liver biopsy. Studies using noninvasive assessment methods (*n* = 5 studies, 10,974 participants) yielded a pooled OR of 1.22 (95% CI: 0.95–1.57, *p* = 0.096), with substantial heterogeneity (*I*^2^ = 69.4%, *τ*^2^ = 0.0304). The test for subgroup differences was not statistically significant (*χ*^2^ = 3.63, df = 1, *p* = 0.0568) and was treated as hypothesis-generating rather than confirmatory.

#### 3.4.2 Subgroup analysis by geographic region

**Figure 5:**
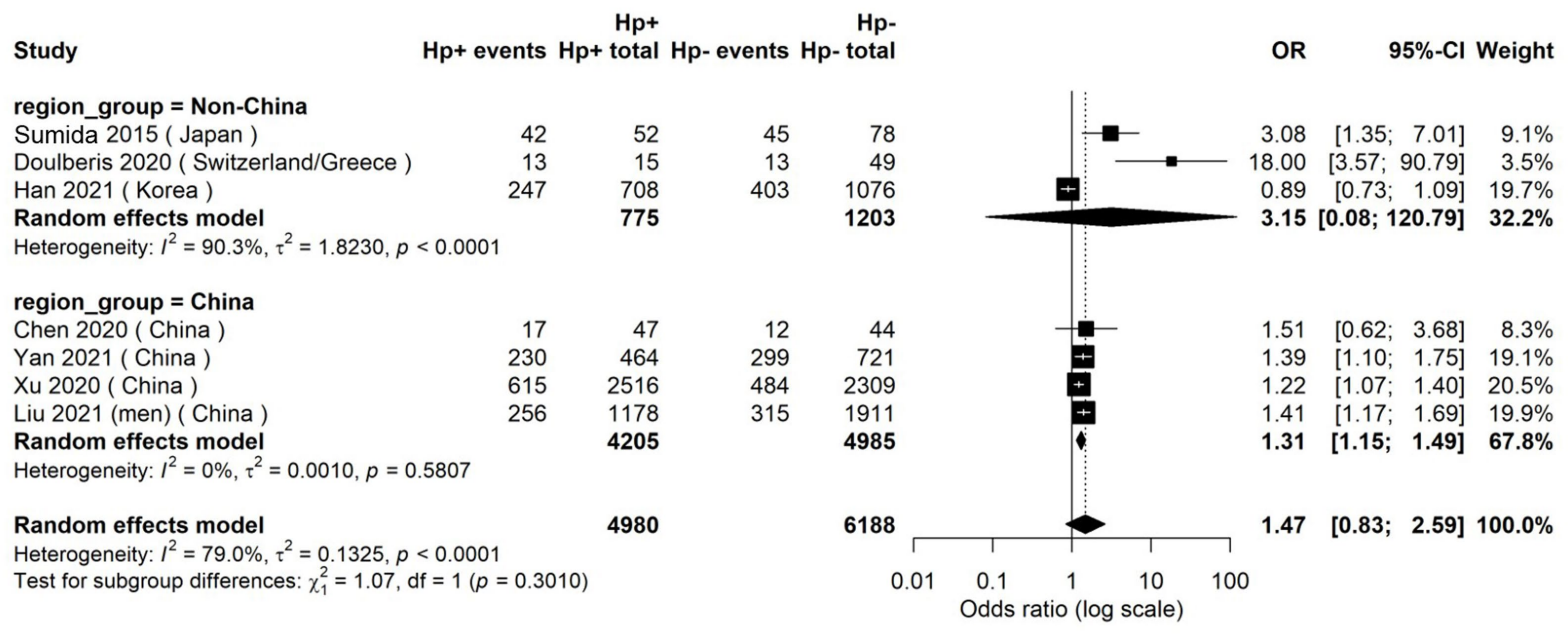
Forest plot of the association between *H. pylori* infection and MAFLD/NAFLD severity stratified by geographic region. Studies were grouped as non-Chinese countries (Japan, Korea, Switzerland/Greece; *n* = 3) versus Chinese studies (*n* = 4). The association was observed only in Chinese studies (OR 1.31, 95% CI 1.15–1.49, I^2^ = 0%), whereas the non-Chinese subgroup was highly imprecise and heterogeneous (I^2^ = 90.3%).

Geographic subgroup analysis revealed that the only statistically significant subgroup finding was observed in Chinese studies (Figure 5). Studies conducted in China (*n* = 4 studies, 9,190 participants) showed a modest association with an OR of 1.31 (95% CI: 1.15–1.49, *p* = 0.007) and no observed heterogeneity (*I*^2^ = 0%). In contrast, studies conducted outside China (Japan, Korea, Switzerland/Greece; *n* = 3 studies, 1,978 participants) showed a larger but highly imprecise pooled estimate (OR = 3.15, 95% CI: 0.08–120.79, *p* = 0.309) with substantial heterogeneity (*I*^2^ = 90.3%, *τ*^2^ = 1.8230). The test for subgroup differences was not statistically significant (*χ*^2^ = 1.07, df = 1, *p* = 0.3010). These findings suggest that geographic region and population characteristics may contribute to heterogeneity, although the non-Chinese estimate is unstable and should not be interpreted as evidence of a stronger association.

### 3.5 Sensitivity analysis

**Figure 6:**
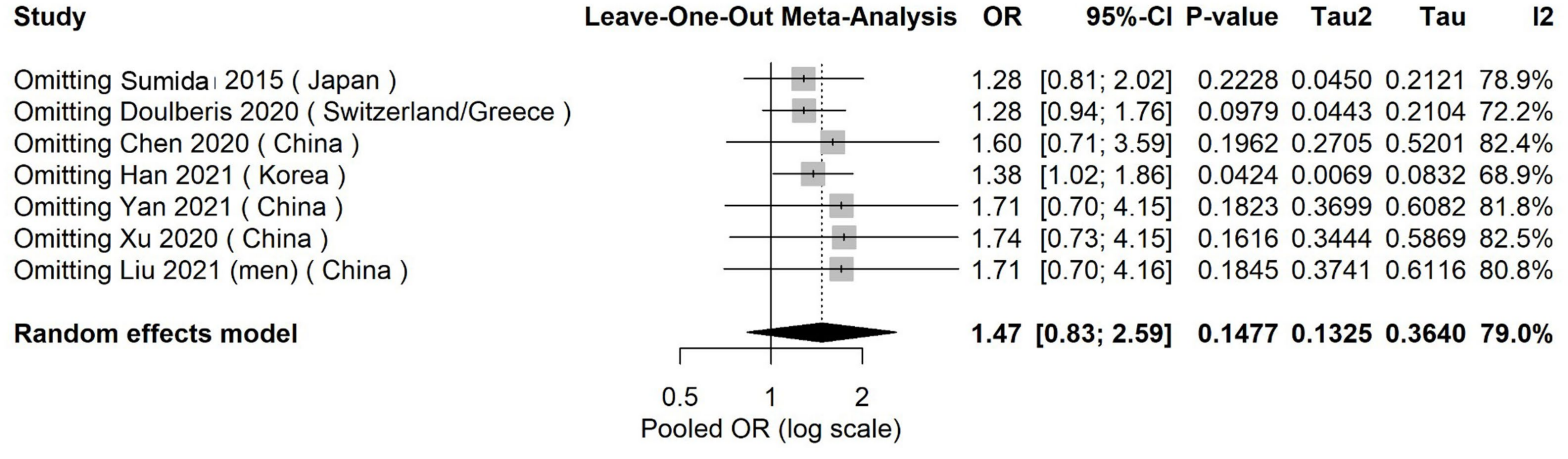
Leave-one-out sensitivity analysis. Each row shows the pooled odds ratio after excluding the indicated study. The analysis indicates that exclusion of individual studies did not materially change the direction of effect, although heterogeneity remained and statistical significance was inconsistent.

A leave-one-out sensitivity analysis was performed to assess whether the direction of effect materially changed after excluding individual studies (Figure 6). Sequential exclusion of individual studies produced pooled ORs ranging from 1.28 (95% CI: 0.81–2.02) when omitting the study by Okushin (2015) to 1.74 (95% CI: 0.73–4.15) when omitting the study by Xu (2020). Omission of the study by Han et al. (22), the only study with an OR-value of <1.0, yielded a pooled OR of 1.38 (95% CI: 1.02–1.86) and reduced heterogeneity to *I*^2^ = 68.9%. This pattern indicates that the study by Han et al. (22) may be linked to between-study heterogeneity, although the analysis did not identify a single study whose exclusion fully resolved heterogeneity. The sensitivity analysis, therefore, supports only a consistent direction of association in most analyses, not a robust statistically significant overall conclusion.

### 3.6 Publication bias

**Figure 7:**
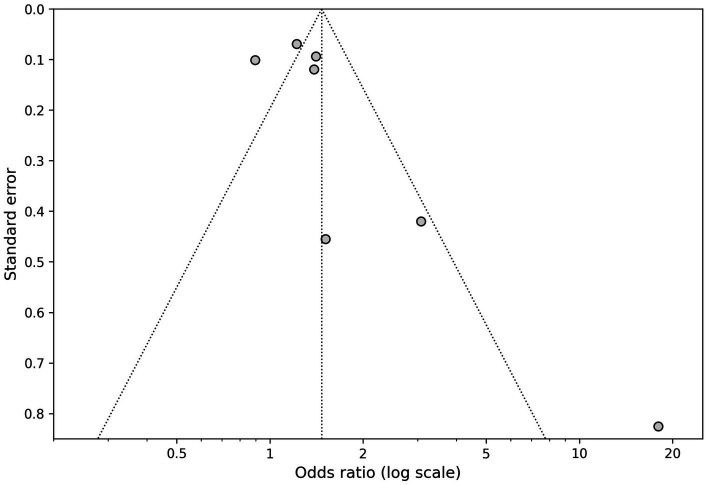
Exploratory funnel plot. The plot shows standard error on the *y*-axis versus odds ratio on a logarithmic *x*-axis. Because only seven studies were included, the plot was not used for formal inference regarding publication bias, and Egger’s test and trim-and-fill were not performed.

The funnel plot was retained only as an exploratory visualization of study size and effect estimates (Figure 7). Because only seven studies were included, formal statistical tests for funnel-plot asymmetry, including Egger’s test, and trim-and-fill methods were not performed. Visual asymmetry appeared to be driven mainly by small biopsy-based studies with large effect estimates and wide CIs, especially the study by Doulberis et al. (20), rather than providing reliable evidence of publication bias.

## 4 Discussion

This systematic review and meta-analysis evaluated the association between *H. pylori* infection and MAFLD/NAFLD severity in seven studies with 11,168 participants. The primary random-effects analysis did not demonstrate a statistically significant overall association (OR = 1.47, 95% CI: 0.83–2.59, *p* = 0.148), and the result was accompanied by substantial heterogeneity (*I*^2^ = 79.0%). The most consistent positive finding was observed in Chinese studies (OR = 1.31, 95% CI: 1.15–1.49, *I*^2^ = 0%), whereas non-Chinese studies and biopsy-based studies were too heterogeneous and imprecise for firm inference. These findings indicate that current data are insufficient to establish a generalisable association between *H. pylori* infection and MAFLD/NAFLD severity.

The biological plausibility of a possible relationship between *H. pylori* infection and MAFLD/NAFLD severity is supported by several mechanisms, although these mechanisms remain inferential in the context of the present meta-analysis. *Helicobacter pylori* infection may be associated with chronic systemic inflammation characterized by elevated pro-inflammatory cytokines and C-reactive protein (26). These inflammatory pathways may be related to hepatic insulin resistance, hepatocellular injury and fibrosis pathways that are central to MASLD/MAFLD progression (27). *Helicobacter pylori* infection has also been linked to altered adipokine profiles, gastric hormone changes and insulin resistance (10, 11). Nevertheless, the present data are observational and cannot determine whether *H. pylori* infection directly affects liver disease severity or is a marker of shared metabolic, socioeconomic, dietary or healthcare-related exposures.

The high heterogeneity observed in the overall analysis warrants cautious interpretation. One major source of heterogeneity was the difference in MAFLD/NAFLD severity assessment. Liver biopsy can define steatohepatitis and fibrosis directly, but biopsy cohorts are usually clinically selected and may have a high pretest probability of advanced disease. In contrast, ultrasound, CAP and liver stiffness measurements are more feasible for population-based studies but capture different aspects of steatosis and fibrosis and are subject to method-specific thresholds. A second source of heterogeneity was variation in *H. pylori* detection methods. Urea breath testing and gastric biopsy primarily identify active infection, whereas serology may reflect previous exposure and may misclassify active infection status. A third source was population heterogeneity, including differences in obesity, diabetes, sex distribution, healthcare setting and regional diet. These factors limit the interpretability of a single overall pooled estimate.

The geographic subgroup analysis should also be interpreted conservatively. The Chinese subgroup showed a modest and statistically significant association with no observed heterogeneity, whereas the non-Chinese subgroup had a larger point estimate but an extremely wide CI and high heterogeneity. This pattern may reflect differences in study design and sample size rather than a true biological difference. Potential biological explanations, such as differences in *H. pylori* virulence factors, host genetic susceptibility and environmental exposures, remain plausible but unproven. East Asian *H. pylori* strains have been reported to differ in virulence factor profiles (28), and MAFLD susceptibility is influenced by genetic and metabolic background (29). However, the present study cannot determine whether strain distribution, genetics or diet modifies the association between *H. pylori* infection and MAFLD/NAFLD severity.

The clinical implications of the observed Chinese subgroup estimate are modest. An OR of approximately 1.31 may be relevant at the population level if confirmed, but it is not sufficient by itself to justify routine *H. pylori* screening or eradication solely to reduce MAFLD/NAFLD severity. Evidence from intervention studies remains mixed. A pilot open-label study reported no long-term effect of eradication on hepatic steatosis but possible improvement in noninvasive fibrosis-related scores (30). Randomized or open-label trials have reported variable findings, including no meaningful improvement in liver fat content in one trial (31), improvement mainly in alanine aminotransferase without consistent metabolic benefit in another trial (32), comparable reduction in steatosis between eradication plus standard therapy and standard therapy alone but greater improvement in homeostatic model assessment of insulin resistance after successful eradication in a pilot study (33) and greater reductions in CAP and inflammatory markers in a more recent randomized trial (34). These trial data suggest biological plausibility but do not provide definitive evidence that eradication prevents progression of MAFLD/NAFLD severity.

Several limitations of this meta-analysis should be acknowledged. First, substantial heterogeneity limits the precision and generalisability of the pooled estimate, and subgroup analyses with few studies should be considered exploratory. Second, all included studies were observational with cross-sectional or case–control designs, precluding conclusions about temporality or causality. Reverse causality cannot be excluded because the time interval between *H. pylori* testing and MAFLD/NAFLD severity assessment was not consistently reported. Third, adjustment for confounders differed across studies, and residual confounding by diet, physical activity, socioeconomic status, medication use, diabetes, obesity and genetic variants remains possible. None of the included studies consistently reported proton pump inhibitor exposure, which may affect *H. pylori* detection and gut microbiome composition, and prior eradication therapy was not reliably reported. Fourth, *H. pylori* detection methods varied across studies; serology may reflect past exposure rather than active infection. Fifth, studies using NAFLD criteria were considered clinically comparable with MAFLD because they were conducted before widespread adoption of the MAFLD nomenclature; however, this may have introduced misclassification due to evolving definitions. Sixth, Japanese- and Korean-language databases were not searched directly, although English-language studies from these countries were captured. Seventh, the review was not registered prospectively, and the small number of studies precluded reliable meta-regression, Egger’s test or trim-and-fill analysis.

Despite these limitations, this study has several strengths. It focuses specifically on MAFLD/NAFLD severity rather than disease prevalence, which is clinically relevant because severity is more closely related to long-term liver outcomes. The search included major international databases and Chinese databases, and duplicate screening, data extraction and quality assessment were performed independently. The analysis also explicitly evaluated the impact of severity assessment method, geographic region and leave-one-out sensitivity on the interpretation of the results.

Future research should include prospective cohort studies with standardized assessment of active *H. pylori* infection and MAFLD/NAFLD severity. Ideally, future studies should record proton pump inhibitor use, prior eradication therapy, antibiotic exposure, metabolic medication use, time interval between exposure and outcome assessment and host genetic and dietary factors. Randomized trials evaluating *H. pylori* eradication should include validated liver endpoints, such as magnetic resonance imaging–proton density fat fraction, CAP, liver stiffness, serum fibrosis markers and, where ethically feasible, paired histology. Such studies are necessary to determine whether *H. pylori* infection is a modifiable factor related to MAFLD/NAFLD progression or merely a marker of shared risk factors.

In conclusion, the current meta-analysis does not demonstrate a statistically significant overall association between *H. pylori* infection and MAFLD/NAFLD severity. A modest association was observed only in Chinese studies, whereas biopsy-based and non-Chinese subgroup findings were imprecise and unstable. The evidence should therefore be interpreted as hypothesis-generating. Additional well-designed prospective studies and randomized trials are needed before *H. pylori* eradication can be considered a strategy for modifying MAFLD/NAFLD severity.

## Data Availability

The original contributions presented in the study are included in the article/supplementary material, further inquiries can be directed to the corresponding author.
